# *Eucommia ulmoides* Extract Attenuates Oxidative Stress, Endoplasmic Reticulum Stress and Apoptosis Induced by Acute Hypoxia via the Nrf2/Keap1 Pathway in Largemouth Bass (*Micropterus salmoides*)

**DOI:** 10.3390/antiox15091207

**Published:** 2026-09-20

**Authors:** Kaiwen You, Yixuan Li, Wanqing Zhong, Jie Yu, Yamei Wu, Yuqi Guo, Haoyang Liu, Haiping Xie, Kai Yuan, Yihong Chen, Lei Wang

**Affiliations:** 1 Institute of Modern Aquaculture Science and Engineering (IMASE), Guangdong Provincial Engineering Technology Research Center for Environmentally-Friendly Aquaculture, Guangdong Provincial Key Laboratory of Insect Developmental Biology and Applied Technology, Institute of Insect Science and Technology, Guangzhou Key Laboratory of Subtropical Biodiversity and Biomonitoring, Key Laboratory of Ecology and Environmental Science in Guangdong Higher Education, School of Life Sciences, South China Normal University, Guangzhou 510631, China; 2024023140@m.scnu.edu.cn (K.Y.); v1499657965@163.com (Y.L.); 13103540704@163.com (W.Z.); 13824976631@163.com (J.Y.); yuqiguo0714@163.com (Y.G.); 2024023370@m.scnu.edu.cn (H.L.); 2 Agro-Tech Extension Center of Guangdong Province, Guangzhou 510000, China; 13924117321@163.com (Y.W.); haiping123@163.com (H.X.); 3 School of Life Science, Huizhou University, Huizhou 516007, China; yk-1256@163.com

**Keywords:** freshwater teleost, hypoxic injury, EUE, antioxidant capacity, TOR, apoptosis

## Abstract

Due to the hypoxic environment caused by global warming and intensive aquaculture process, fish are susceptible to oxidative damage and death. *Eucommia ulmoides* extract (EUE) has demonstrated antioxidant and anti-inflammatory properties. However, whether EUE can mitigate hypoxia-induced negative effects remains uninvestigated in fish. This study investigated the effect of 0 and 1.0 g/kg EUE on acute hypoxia-induced damage in largemouth bass and its potential mechanisms. The results showed that EUE alleviated the deterioration of plasma activities (ALT and AST) induced by acute hypoxic stress, decreased the number of macrophages and increased the total number of blood cells. EUE was effective in reducing the level of apoptosis and the content of ROS, Ca^2+^, and NO in blood cells. Additionally, EUE alleviated liver tissue injury caused by acute hypoxic stress, which could be associated with increased total antioxidant capacity and antioxidant enzyme activities (SOD, CAT and GSH-Px). Furthermore, EUE up-regulated the expression levels of *Nrf2* and *CAT*, activated *XBP1* and *IRE* expression and down-regulated *GRP78* expression, relieving the endoplasmic reticulum (ER) stress response. The down-regulation of *Caspase3* and *Caspase9* is evidence that EUE inhibited apoptosis in acute hypoxic stress. In summary, EUE reduced oxidative damage, ER stress, and apoptosis caused by hypoxic stress via the Nrf2/Keap1 signaling pathway in largemouth bass. This study presents a part of the theoretical foundation for the EUE to resist damage caused by hypoxic stress, thereby establishing a basis for sustainable development in aquaculture.

## 1. Introduction

Due to various factors such as global climate change, eutrophication, and human activities, the phenomenon of low dissolved oxygen (DO) in water has become increasingly severe in recent years [1]. In some tropical regions, the concentration of oxygen has even declined by up to 40% over the past 50 years. In addition to natural water bodies, high-density aquaculture may also lead to short-term hypoxia hazards in aquaculture water bodies. The concentration of dissolved oxygen in water is an essential factor affecting the growth, development, metabolism and energy transfer of aquatic organisms [2]. Under low-DO conditions, the activity of reactive oxygen species (ROS) and oxidative stress increases sharply, leading to damage to proteins, DNA, and lipids, endoplasmic reticulum (ER) stress [3], apoptosis [4], and tissue damage. In response to low oxygen stress, the organism produces a series of changes in metabolism-related molecules to adapt to the stress.

ROS is a natural byproduct of oxygen metabolism in mitochondria. Normal levels of ROS in cells are beneficial for promoting cellular metabolism and signal transduction, maintaining cellular homeostasis. However, various physical and chemical stressors in the external environment, such as hypoxia, could lead to the release of large amounts of ROS [5]. When there is an excess of ROS, it can cause oxidative stress in cells. In order to maintain oxidative balance, cells will regulate the production of antioxidants to eliminate ROS. Aquatic organisms, like other animals, had developed an antioxidant defense system, which could clear ROS and resist oxidative damage caused by stress [6]. Hypoxia promoted the antioxidant enzyme system’s response, as reported in rainbow trout (*Oncorhynchus mykiss*) [7] and white shrimp (*Penaeus vannamei*) [8], which enhanced antioxidant capacity to cope with hypoxic stress. Nrf2 is a key transcription factor involved in regulating antioxidant responses. Under oxidative stress, Nrf2 dissociated from Keap1 and entered the nucleus to promote the transcription of cis-acting elements, such as up-regulation antioxidant enzymes, to remove ROS. Most studies have shown that the Nrf2/Keap1 signaling pathway can effectively regulate oxidative damage [4,9,10]. Additionally, the activation of the PI3K/AKT/TOR signaling pathway can also effectively promote antioxidant effect [11,12]. In summary, enhancing the organism’s antioxidant capacity through activating the Nrf2/Keap1 pathway and PI3K/AKT/TOR signaling pathway could resist changes in the external physical and chemical environment.

Hypoxia is closely related to ER stress. Increasing evidence suggests that oxidative stress and Ca^2+^ imbalance can lead to ER stress [13]. The accumulation of ROS caused by hypoxia triggers ER stress and activates the unfolded protein response (UPR), which restores the correct folding and transport function of proteins in the endoplasmic reticulum cavity [14,15]. However, if ER stress response is too strong or lasts too long, it will up-regulate the expression of *CHOP* and the cysteine protease family caspases, thereby initiating the cell apoptosis program [16]. In studies on grass carp (*Ctenopharyngodon idella*) [17], it was found that when exposed to bisphenol, the activation of the UPR signaling pathway in liver induces the activation of the downstream BCL2 protein family and promotes apoptosis. Cell apoptosis is an active death process that cells undergo to better adapt to the living environment [18]. The occurrence of cell apoptosis is often accompanied by external stimuli such as high temperature, cold, and hypoxia [19], and is regulated by various apoptosis-related genes [20]. Current studies show that adverse external environment can activate the expression of apoptosis-related genes, such as *Caspases* [21], by inducing oxidative stress and activating the UPR signaling pathway to promote the process of apoptosis [22]. Hypoxic stress greatly increased the activity of caspase3, caspase8, and caspase9 in the gills of grass carp, indicating that hypoxia may mediate apoptosis through an exogenous pathway [16]. These results indicate that hypoxia trigger the production of oxidative stress in cells, which leads to the endoplasmic reticulum stress response. The endoplasmic reticulum activates the UPR protection mechanism, and the endoplasmic reticulum stress can further induce the occurrence of apoptosis.

Furthermore, combined with the previous research results of our previous result, hypoxia can lead to a decrease in the total number of blood cells and morphological damage of blood cells in largemouth bass [23]. Flow cytometry analysis confirmed that the apoptosis rate, Ca^2+^ level, NO production and ROS of blood cells were significantly increased under hypoxic stress. In addition, we investigated the expression of genes involved in oxidative stress regulation. The results showed that under hypoxic conditions, both the *Nrf2/Keap1* signaling pathway and the oxidative stress regulator *Hif-α* exhibited significant up-regulation. The expression of catalase (*CAT*) and glutathione peroxidase (*GPx*) were enhanced. Simultaneously, the Hippo signaling pathway, which regulates apoptosis, was activated under hypoxic conditions, thereby inducing cellular death [23].

Therefore, in order to avoid oxidative stress and apoptosis in blood cells of largemouth bass caused by hypoxia, researchers are committed to explore safe and reliable methods to alleviate hypoxia suitable for aquaculture environment. Plant extracts are rich in active ingredients and many studies have proved that some plant extracts have antibacterial [24], anti-inflammatory [25] and antioxidant functions, which could be added as core components to play a role in drugs [26]. EUE is a unique Chinese medicinal herb and its main active ingredients include phenols, steroids, terpenoids and flavonoids [27]. These ingredients endow EUE with various biological functions, such as promoting growth, regulating blood sugar and blood lipids, regulating immunity, and antioxidation [28,29]. Currently, EUE is mainly used in the breeding process of livestock and poultry which increased the weight of pigs [30] and lambs [27], and enhanced the antioxidant capacity of meat chickens during heat stress [31]. Furthermore, chlorogenic acid, as a core bioactive component of EUE, has been reported to attenuate oxidative stress in laying hens by regulating Nrf2 pathway-related genes and increasing hepatic antioxidant enzyme activities, as well as by reducing hepatic inflammatory responses and apoptosis through activation of PI3K/AKT pathway-related genes and proteins [32]. Previous studies have demonstrated that EUE exerts antioxidant and anti-apoptotic effects in various animal models. For instance, in cultured microglial cells in vitro, EUE was shown to alleviate lipopolysaccharide (LPS)-induced inflammatory responses through the activation of the Nrf2 signaling pathway [33]. In mice, EUE has also been confirmed to alleviate oxidative stress via the Nrf2/HO-1 pathway [34]. In hyperlipidemic rats, EUE administration significantly reduced body weight, lipid accumulation, and serum levels of total cholesterol (TC), triglycerides (TG), and low-density lipoprotein cholesterol (LDL-C), while increasing high-density lipoprotein cholesterol (HDL-C) levels [35]. In aquatic organisms, appropriate dietary EUE can improve the growth performance and disease resistance of channel catfish (*Ictalurus punctatus*) [36]. It has been proven to enhance the antioxidant capacity of fish and protect largemouth bass (*Micropterus salmoides*) from liver damage caused by a high-starch diet [37]. EUE is increasingly used in aquaculture, but its regulatory mechanism for resisting external environmental factors deserves further exploration.

The largemouth bass belongs to the family *Centrarchidae* and the genus *Micropterus*. It is a eurythermal freshwater fish with a survival temperature range of 1–36 °C and an optimal growth temperature range of 20–25 °C; feeding is impaired below 15 °C or above 28 °C [38,39]. Due to its short growth cycle, strong adaptability, and delicious flesh, it has become one of the high-quality freshwater fish species worldwide and also one of the most produced aquaculture species. In terms of dissolved oxygen requirements, the optimal level for growth and physiological maintenance is ≥8.20 mg/L [40]; growth and health are affected when DO falls below 5.50 mg/L, and significant physiological stress occurs at 5.50–1.20 mg/L [41,42,43]. Critical danger occurs at 1.20–0.80 mg/L [44,45,46], and mortality is observed below 0.80 mg/L, with the asphyxiation point reported at 0.33–0.40 mg/L [47]. During the current intensive farming process, hypoxia, as a typical environmental stressor, can inhibit fish growth performance, immune function, and flesh quality by inducing oxidative stress damage and disrupting cellular homeostasis; largemouth bass is prone to die due to hypoxia. EUE is rich in various bioactive compounds including flavonoids, chlorogenic acid, and polysaccharides, and has been demonstrated to possess antioxidant, anti-inflammatory, anti-hypoxic, and hepatoprotective properties. Accordingly, we hypothesized that EUE could mitigate the physiological damage induced by acute hypoxia in largemouth bass by modulating the antioxidant defense system, endoplasmic reticulum (ER) stress, and apoptosis-related pathways. To test this hypothesis, we first supplemented EUE in the diet of largemouth bass for an 8-week feeding trial, followed by acute hypoxic exposure, with the aim of systematically evaluating the regulatory effects of EUE on antioxidant capacity, ER stress, apoptosis, and their related signaling pathways (such as the Nrf2 antioxidant pathway, ER stress-related pathways, and apoptotic pathways) in largemouth bass under acute hypoxia. This study made the first attempt to evaluate the effects of EUE on ER stress and the apoptosis of largemouth bass during acute hypoxia. We believe that this study will provide a partial theoretical basis for the application of EUE as a feed additive to alleviate hypoxic stress injury in fish, and offer a scientific reference for the sustainable development of aquaculture.

## 2. Materials and Methods

### 2.1. Ethical Statement

Procedures related to animal experimental were strictly in accordance with the Regulations for Animal Experimentation of South China Normal University. This study was approved by the Scientific Research Ethics Committee of South China Normal University (Approval number: SCNU-SLS-2024-011).

### 2.2. Experimental Diets

Two isonitrogenous and isolipidic diets were formulated using fish meal, soybean meal, and soybean protein concentrate as the primary protein sources, and fish oil and soybean oil as the lipid sources. The basal diet was supplemented with either 0 or 1 g/kg EUE (*Eucommia ulmoides* extract, containing 15% polysaccharides, 5% chlorogenic acid, and 9% flavonoids, provided by Guangdong Agricultural Technology Extension Center) to yield the Control group and EUE group, respectively. The formulation and proximate composition of the diets are presented in Table 1. All dry ingredients were ground and passed through a 40-mesh sieve, then thoroughly mixed in a food mixer for 30 min. Fish oil and soybean oil were then added to the dry mixture and blended homogenously. Subsequently, an appropriate amount of water was added to obtain a homogeneous dough. The dough was then pelletized using a pelletizer (F-26, South China University of Technology, Guangzhou, China) equipped with a 2.0 mm die. The resulting pellets were oven-dried at 60 °C for 24 h, cooled to room temperature, and stored at −20 °C until use [48].

### 2.3. Experimental Fish and Feeding Management

All experimental largemouth bass were obtained from the Panyu Aquaculture Base of South China Normal University. Prior to the feeding trial, the fish were cultured in outdoor cages and fed the control diet for two weeks to acclimate them to the experimental diet and environmental conditions. The average body length and body weight of the largemouth bass were 7.32 ± 0.56 cm and 27.27 ± 0.19 g, respectively. After being starved for 24 h, the fish were randomly assigned to two groups: the Control group and the EUE group. Each group consisted of three replicate cages (1.0 m × 1.0 m × 1.0 m), with 30 fish stocked per cage. During the trial, the photoperiod was maintained at 12 h light and 12 h dark. The experimental fish were fed to apparent satiety at 07: 00 and 17:00 for 8 weeks. Daily feed consumption, as well as the number and weight of dead fish, were recorded for each cage. Continuous aeration was provided to each cage via air stones to maintain dissolved oxygen levels. The rearing water consisted of aerated tap water; uneaten feed and feces were removed daily, and approximately one-third of the water volume was renewed daily. The water quality indicators were detected by YSI ProQuatro Multiparameter Meter (YSI Incorporated, Yellow Springs, USA) daily (pH 7.6 ± 0.2, temperature 25 ± 1 °C, ammonia nitrogen ≤ 0.05 mg/L, and DO ≥ 6.5 mg/L).

### 2.4. Hypoxic Stress Test

**Figure 1:**
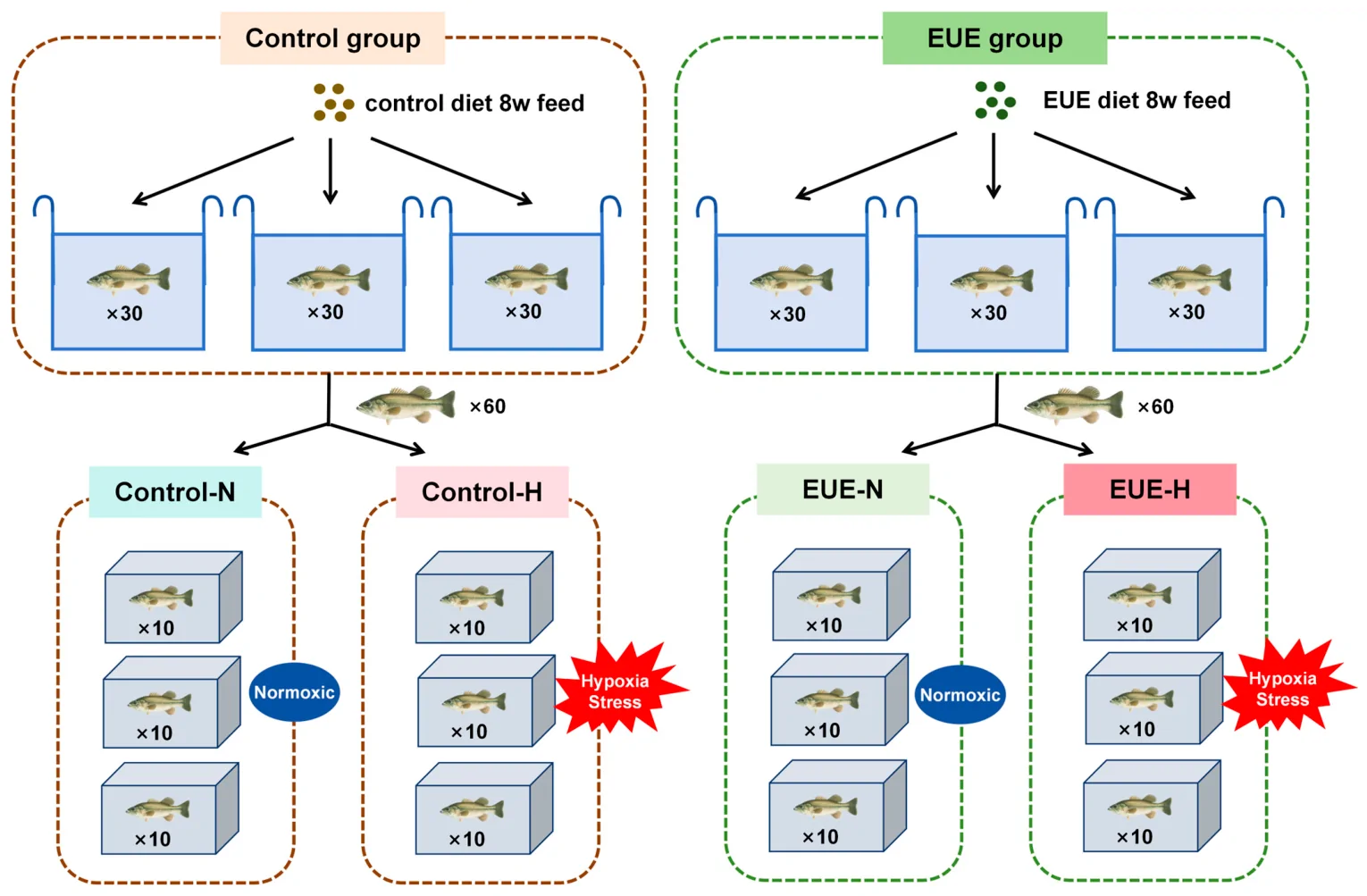
Schematic diagram of experimental groups for feeding conditions and hypoxic stress treatment.

Following an 8-week feeding period, 60 healthy and uniformly sized largemouth bass (average weight: 118.64 ± 8.13 g) were randomly selected from each of the Control and EUE groups. Subsequently, 12 plastic aquaria (50 cm × 35 cm × 30 cm, effective water volume of 52 L) were prepared, with six aquaria allocated to each of the Control and EUE groups for the subsequent hypoxic stress experiment. All aquaria were maintained at a constant water temperature of 25 °C. The 60 fish from each group were randomly assigned to six plastic aquaria at a density of 10 fish per aquarium, and the biomass density in each aquarium was 22.8 kg/m^3^. Hypoxic challenge was then conducted under four treatment combinations based on oxygen regimen and dietary intervention: (1) normoxic Control (Control-N), (2) normoxic EUE supplementation (EUE-N), (3) hypoxic Control (Control-H), and (4) hypoxic EUE supplementation (EUE-H). Each group had three aquaria (Figure 1). The normoxic groups (Control-N and EUE-N) were maintained at a dissolved oxygen (DO) concentration of approximately 6.50 mg/L. To induce acute hypoxic stress, water recirculation and aeration were terminated in the hypoxic groups (Control-H and EUE H), and the aquaria were sealed, following our previously established hypoxia exposure protocol [23]. During the hypoxic induction phase, dissolved oxygen (DO) concentrations were recorded at 5 min intervals using a portable DO meter. The DO concentration declined from 6.50 mg/L to the target level of 3.25 mg/L over approximately 90 min solely through the respiratory consumption of the fish. Once the target DO concentration (3.25 mg/L) was reached, the fish in the hypoxic groups were maintained at this level for an additional 40 min prior to sampling.

### 2.5. Sample Collection and Disposition of Fish

Before sampling, the fish were anesthetized with MS-222 solution (Aladdin, Shanghai, China) [52]. Based on the results of preliminary experiments, the anesthetic concentration of MS-222 was determined to be 70 mg/L, and the solution was freshly prepared with water from the rearing system immediately before use. Fish were placed in the anesthetic solution and their behavioral changes were closely monitored. After approximately 60 s of anesthesia, when the fish lost equilibrium, exhibited slow swimming, reduced opercular movement, and showed no response to external stimuli, they were rapidly removed from the solution for subsequent sampling [53,54]. Three fish were randomly chosen from each aquaria. Blood was withdrawn from the caudal vein, and centrifuged (2000 rpm at 4 °C) for 10 min. The plasma was taken for the determination of biochemical parameters. And the blood cell was taken for flow cytometry. The liver tissues were dissected on ice, with one part stored in 4% paraformaldehyde solution (Biosharp, Hefei, China) for making slices, one part stored at −80 °C for the determination of chemical and biochemical analyses, and the other part immediately put into liquid nitrogen and stored at −80 °C for RNA extraction. Tissue samples from the three fish in each aquarium were pooled into one mixed sample, and placed in a single EP tube; all subsequent procedures were performed using this pooled sample. Each aquarium was treated as an independent replicate (n = 3 per treatment) for statistical analysis. After the completion of the feeding trial and all sampling procedures, all remaining experimental fish were euthanized by immersion in a buffered MS-222 solution at a concentration of 500 mg/L for a minimum of 30 min [55]. All carcasses were collected, placed in designated biohazard bags, and stored in a freezer at −20 °C. The carcasses were then reported to the Laboratory Safety Department of South China Normal University and disposed of as biological waste by incineration through a licensed waste disposal service.

### 2.6. Biochemical Analysis of Plasma

The total protein (TP) content in plasma was measured using the total protein assay kit (Haifei Biotechnology Co., Ltd., Xiamen, China, P/N: 105-015579-00). Total cholesterol (TC) was measured in plasma using a total cholesterol assay kit (Shanghai Gaochuang Chemical Technology Co., Ltd., Shanghai, China, Cat. No F002-1). Total glucose (TG) content in plasma was determined by glucose determination reagent (Biostest, Beijing, China, Cat. No. K-FRUGL). Triglyceride (TG) content in plasma was determined by the Triglyceride (TG) Content Assay kit (Boxbio, Beijing, China, Cat. No. AKFA003M). The activity of glutamic pyruvic transaminase (ALT) and glutamic oxaloacetic transaminase (AST) was, respectively, measured in four groups of largemouth black bass plasma by ELISA kits were obtained from COIBO BIO Co., Ltd. (Shanghai, China, Cat. No. CB10022-Fi; CB10037-Nu). All operations were strictly in accordance with the kit instructions. The levels of above plasma indicators were analyzed by an Automatic Biochemical Analyser (CHEMIX800, Kobe, Japan).

### 2.7. Giemsa Staining and Total Blood Cell Count

A 10 µL untreated blood sample was stained with 20 µL of Giemsa A solution (Biosharp, Hefei, China) at room temperature for 0.5 min, followed by gentle mixing with 40 µL of Reisch Jimsa B solution (Biosharp, Anhui, China) for 1 min. The prepared blood smear was rinsed with distilled water to remove the dye. Finally, it was observed with a polarized light microscope (Leica Microsystems, Wetzlar, Germany) and photographed after drying. At the same time, the blood cells after multiple dilutions were counted on the hemocytometer under the microscope (Leica Microsystems, Wetzlar, Germany).

### 2.8. Detecting Indicators of Cell Apoptosis, Nitric Oxide, Ca^2+^, and ROS by Flow Cytometry

The treatment and flurescence staining of the blood cells were conduted performing our previous method. Annexin V-FITC/PI Fluorescence double staining apoptosis assay kit (Elabscience Biotechnology Co., Ltd., Wuhan, China) was used to stain the cells for the detection of cell apoptosis. Intracellular ROS was estimated by using a fluorescent probe, DCFH-DA (Dalian Meilun Biotechnology Co., Ltd., Dalian, China). Fluo-3 and AM ester (eBioscience Biotechnology, San Diego, CA, USA) were used to stain the cells or measurement of Ca^2+^ level. DAF-FM DA (Dalian Meilun Biotechnology Co., Ltd., Dalian, Liaoning, China) was used to stain the cells for measurement of NO. After staining, the blood cells were analyzed with a BD FACS Aria III flow cytometer (BD, San Jose, CA, USA).

### 2.9. Histology Observation

The liver tissues were dehydrated in alcohol and embedded in paraffin wax. Then, the tissues were serially sectioned and stained with hematoxylin and eosin (H & E) [56]. The liver morphological was observed using a polarized light microscope (Leica, Wetzlar, Germany). The Image J Launcher (Version 1.54s, NIH, Bethesda, MD, USA) software was used to identify myofiber characteristics.

### 2.10. Measurement of Antioxidant Parameters

Total antioxidant capacity (T–AOC), malondialdehyde (MDA) and the activity of SOD, catalase (CAT), glutathione peroxidase (GSH-Px) in liver were determined using the reagent kit (Nanjing Jiancheng Bioengineering Institute, Nanjing, China).

### 2.11. Tissue Total RNA Extraction and qRT-PCR

A rapid RNA extraction kit (HaiGene Biotech Co., Ltd., Harbin, China, Cat. No. B0132) was used to isolate the total RNA samples from four groups of liver. A total of 1% agarose gelelectrophoresis and Nanodrop one (Thermo, Waltham, MA, USA) were used to assay RNA purity and concentration, respectively. cDNA synthesis was performed using a PrimeScript^TM^ RT reagent Kit (Takara, Dalian, China, Cat. No. RR036A) from 2 μg total RNA. Then, 100 ng of cDNA in 10 µL reaction was used as a template for qRT-PCR. qRT-PCR was performed on a CFX96 Real-Time PCR System (Bio-Rad, Hercules, CA, US) with SYBR Green (YUANZAN, Kunshan, China, Cat. No. 86AF02). PCR conditions were; initial denaturation at 95 °C for 3 min, and 39 cycles of 95 °C for 10 s, Tm (52–60) °C for 30 s, 72 °C for 45 s. From three independent biological replicates, three technical replicates from each were carried for qRT-PCR According to the preliminary experiment, β-actin was chosen as the reference gene. Sequences of primer for qPCR were listed in Table S1. The relative gene expression was analyzed using the 2^−∆∆CT^ method [57].

### 2.12. Statistical Analysis

Statistical analyses were performed using SPSS version 26.0 (IBM Corp., Armonk, NY, USA). Data on plasma metabolites and antioxidant parameters were analyzed by one-way analysis of variance (ANOVA), followed by Fisher’s least significant difference (LSD) post hoc test for multiple comparisons [58]. Total hemocyte counts, flow cytometry results, and gene expression data were first assessed for normality using the Shapiro–Wilk test, and then analyzed by two-way ANOVA with oxygen regime (normoxia vs. hypoxia) and dietary treatment (Control vs. EUE) as fixed factors [59]. All data are presented as mean ± standard deviation (SD), with each aquarium considered as an independent replicate (n = 3 per treatment group).

## 3. Results

### 3.1. Effects of EUE Under Hypoxic Stress on Plasma Biochemical Measurements

The plasma biochemical parameters of largemouth bass after hypoxic stress are presented in Table 2. No significant differences in total protein (TP) levels were observed among the Control-N, Control-H, and EUE-H groups (*p* > 0.05), whereas the TP concentration in the EUE-N group was significantly elevated compared to the aforementioned three groups (*p* < 0.05). Regarding glucose (GLU), the concentration in Control-H group was significantly lower than that in the other groups (*p* < 0.05), whereas no significant difference was found between EUE-N and EUE-H (*p* > 0.05). Hypoxic stress induced a marked overall reduction in triglyceride (TG) levels (*p* < 0.05); however, the TG level in the EUE-H group was significantly higher than that in the Control-H group (*p* < 0.05). Cholesterol (CHOL) levels did not vary significantly among all groups (*p* > 0.05). Notably, EUE addition significantly reduced the activities of ALT and AST, as evidenced by significantly lower values in the EUE-N group compared to the Control-N group, and in the EUE-H group compared to the Control-H group (*p* < 0.05); moreover, the EUE-N group exhibited the lowest enzyme activities among all treatments.

### 3.2. Effects of EUE Under Hypoxic Stress on Total Blood Cell Count

**Figure 2:**
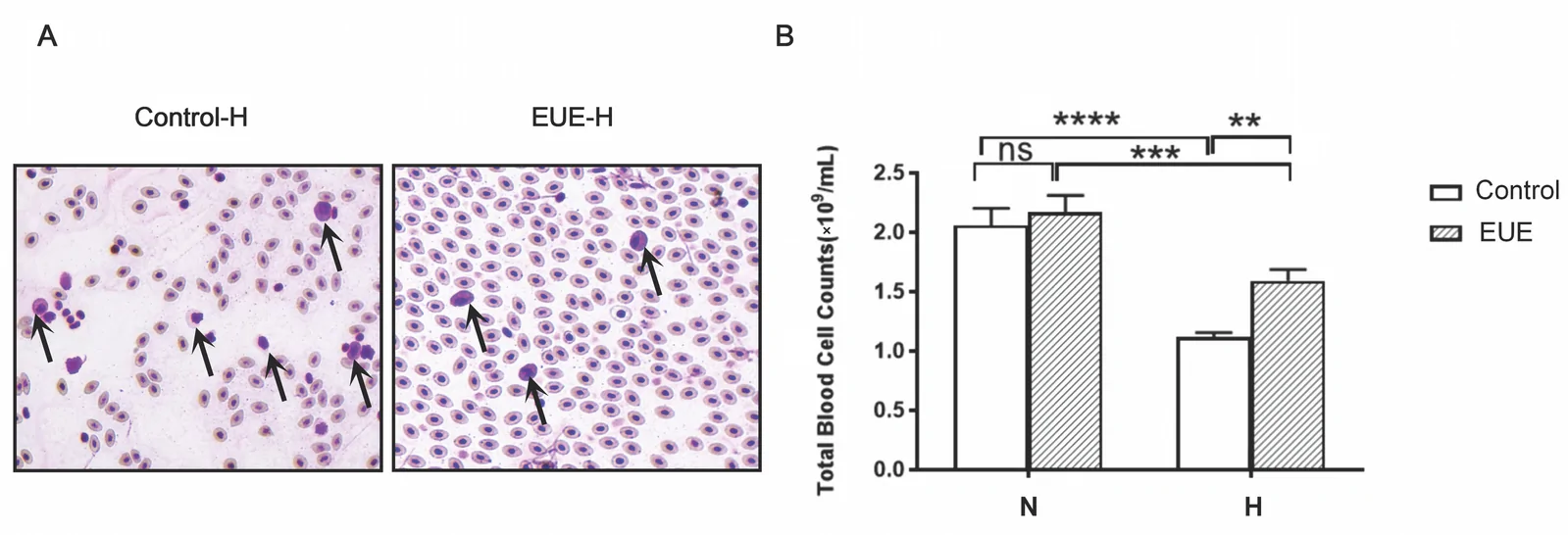
The blood cell morphology (**A**) and total blood cell count (**B**) of largemouth bass in normal and hypoxic groups after feeding with EUE. Macrophages are marked with arrows. N, normoxia; H, hypoxia. ns indicates no significant difference (*p* > 0.05); the **, *** or **** at the top of the bar chart indicates statistically significant; **, *** or **** indicate extremely significant difference (*p* < 0.01, *p* < 0.005 or *p* < 0.001). Data are presented as mean ± SD (n = 3, two-way analysis of variance).

The Giemsa staining results (Figure 2A) clearly indicated that the total blood cell in the Control-H group was decreased relative to the EUE-H group, while macrophage-like cells exhibited the opposite trend. Statistical analysis revealed that under normoxic conditions, no significant difference in total blood cell count (TBCC) was observed between the Control-N and EUE-N groups (*p* > 0.05) (Figure 2B). However, hypoxic stress induced a decreasing trend in the TBCC of largemouth bass. Specifically, TBCC in both the Control-H and EUE-H groups were significantly lower than those of Control-N and EUE-N groups (*p* < 0.001 and *p* < 0.005). Notably, TBCC in the EUE-H group was significantly higher than that in the Control-H group (*p* < 0.01). Furthermore, the results of the Control-H and EUE-H groups exhibited a trend consistent with the Giemsa staining observations.

### 3.3. Effects of EUE Under Hypoxic Stress on Apoptosis of the Blood Cell

**Figure 3:**
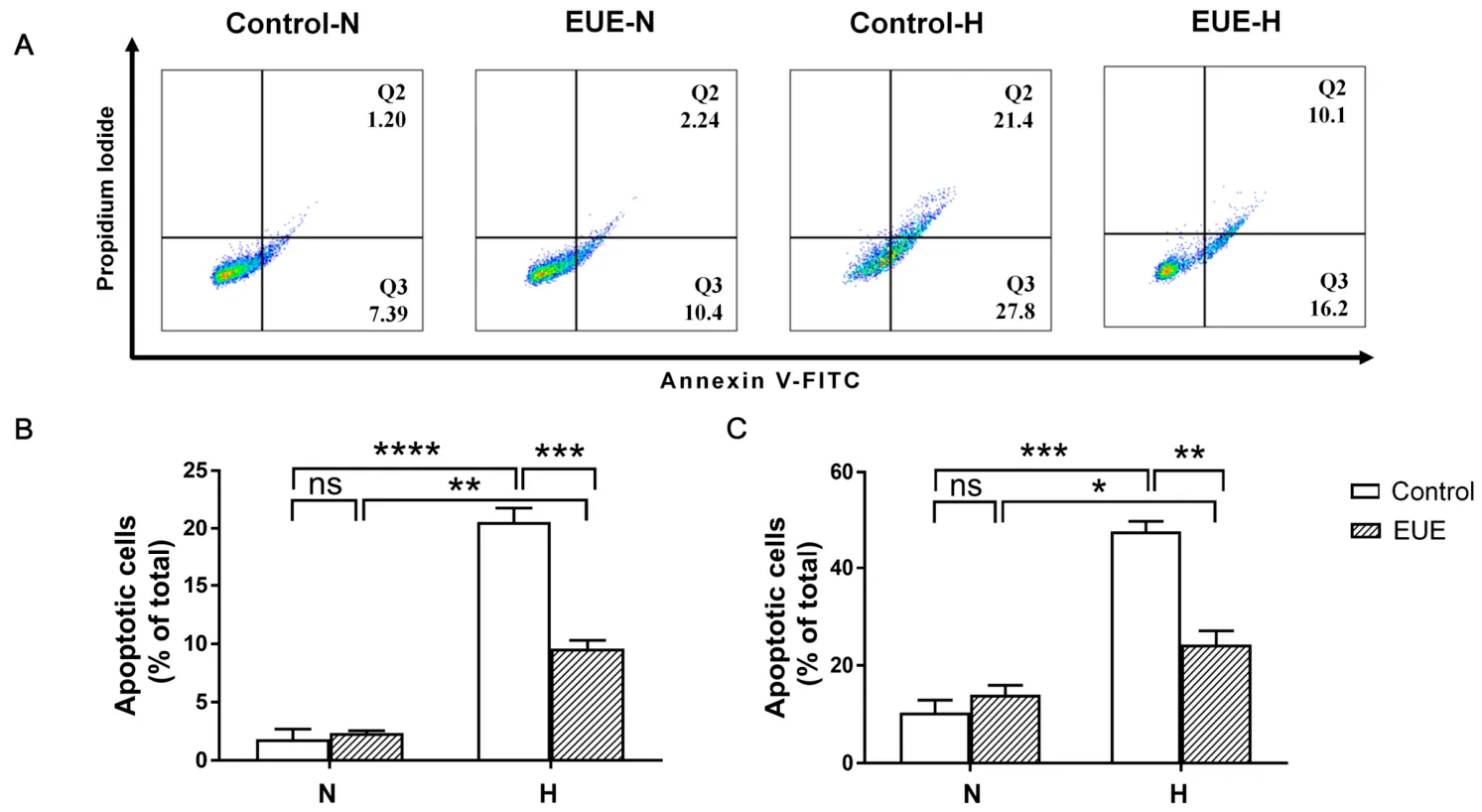
The apoptosis levels of blood cell in normal and hypoxic groups after feeding with EUE. (**A**) Quantitative results of flow cytometry in blood cells. Q1 quadrant represents unviable cells, Q2 quadrant represents cells that are in late apoptosis, Q3 quadrant represents cells that are in early apoptosis, and Q4 represents viable cells. (**B**) Bar graph of late apoptotic cells ratio in flow cytometry (Q2). (**C**) Bar graph of average apoptotic cells ratio in flow cytometry (Q2 + Q3). N, normoxia; H, hypoxia. ns indicates no significant difference (*p* > 0.05); the *, **, *** or **** at the top of the bar chart indicates statistically significant; * shows significant difference (*p* < 0.05); **, *** or **** indicate extremely significant difference (*p* < 0.01, *p* < 0.005 or *p* < 0.001). Data are presented as mean ± SD (n = 3, two-way analysis of variance).

Compared to the normoxia group, cells were shifted towards the Q2 and Q3 quadrants by hypoxic stress (Figure 3A). Among them, the early (Q3) and late (Q2) apoptotic cells in the Control-H group reached the highest level, surpassing the other groups. Flowjo further quantified these apoptotic cells; the percentage of the late apoptotic cells in the Control-H and EUE-H groups were 20.55% and 9.59% (Figure 3B), which were extremely markedly increased relative to the Control-N and EUE-N groups (*p* < 0.001, *p* < 0.01). It is noteworthy that under hypoxic conditions, the level of late apoptotic cells in the EUE-H group was profoundly significantly lower than the Control-H group (*p* < 0.005). Additionally, the number of late and early apoptotic cells (Q2 + Q3) in Figure 3C showed a similar trend as depicted in Figure 3B.

### 3.4. Effects of EUE Under Hypoxic Stress on ROS, Ca^2+^ and NO of the Blood Cell

**Figure 4:**
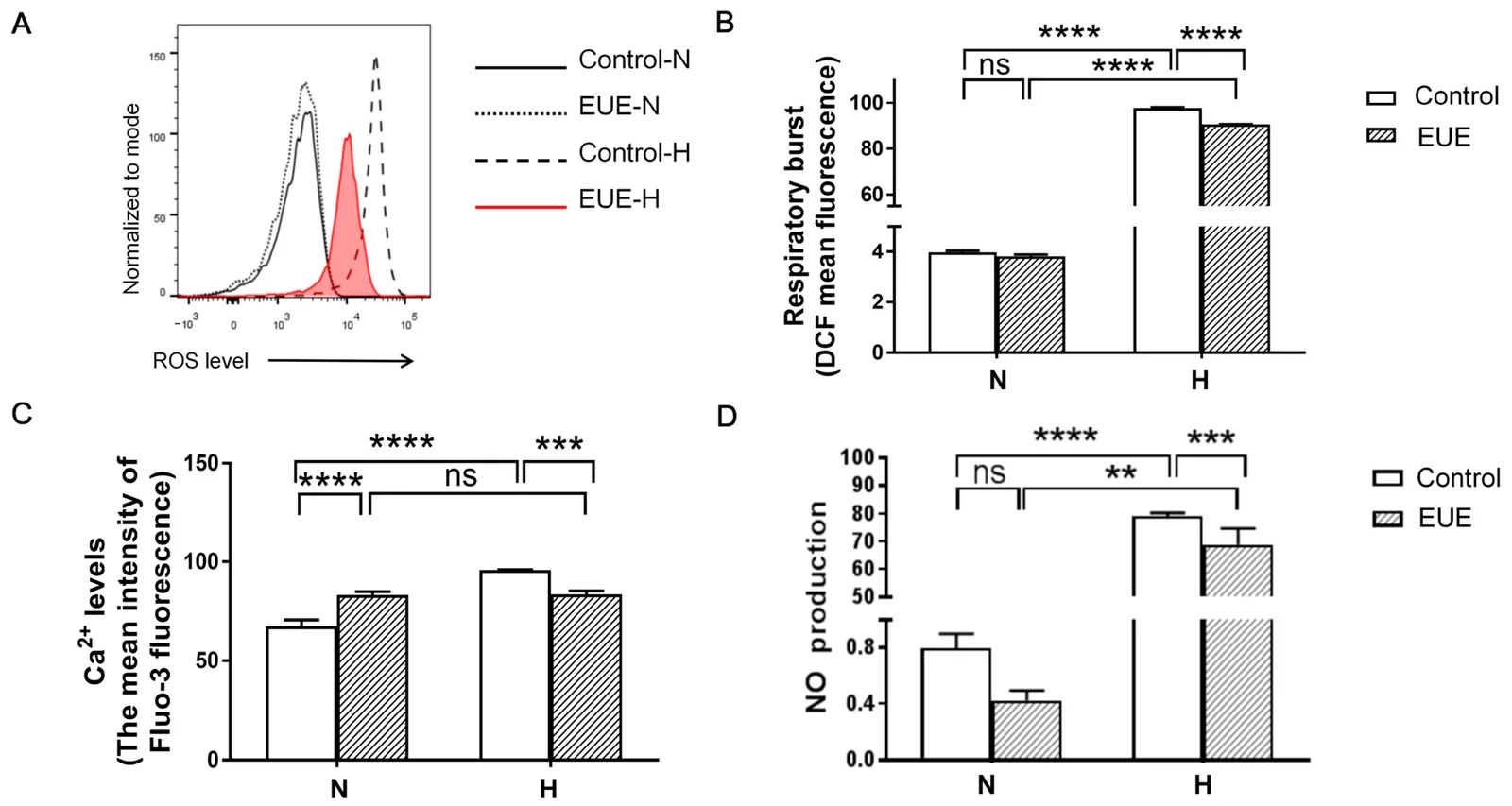
The concentration of ROS (**A**,**B**), Ca^2+^ (**C**) and NO (**D**) of blood cell in normal and hypoxic groups after feeding with EUE. N, normoxia; H, hypoxia. ns indicates no significant difference (*p* > 0.05); the **, *** or **** at the top of the bar chart indicates statistically significant; **, *** or **** indicate extremely significant difference (*p* < 0.01, *p* < 0.005 or *p* < 0.001). Data are presented as mean ± SD (n = 3, two—way analysis of variance).

As shown in Figure 4A,B, hypoxic stress led to an increase in ROS content, as demonstrated by the ROS content in the Control-H and EUE-H groups being 24.60-fold and 23.70-fold higher than the Control-N and EUE-N groups, respectively. Furthermore, although there was no significant change in the Ca^2+^ level between the EUE-N and EUE-H groups (*p* > 0.05, Figure 4C), hypoxia highly and significantly increased Ca^2+^ level in the Control-H group (*p* < 0.001). Also, there is no significant difference between the Control-N and EUE-N groups in NO concentration (*p* > 0.05, Figure 4D). After hypoxic stress, the level of NO production in the Control-H (79.03 ± 1.01%) and EUE-H groups (68.87 ± 6.85%) were sharply increased compared to the normoxia groups (*p* < 0.001, *p* < 0.01). It is interesting that the levels of ROS, Ca^2+^, and NO in the EUE-H group were exceptionally and significantly lower than those in the Control-H group.

### 3.5. Effects of EUE Under Hypoxic Stress on Liver Morphology

**Figure 5:**
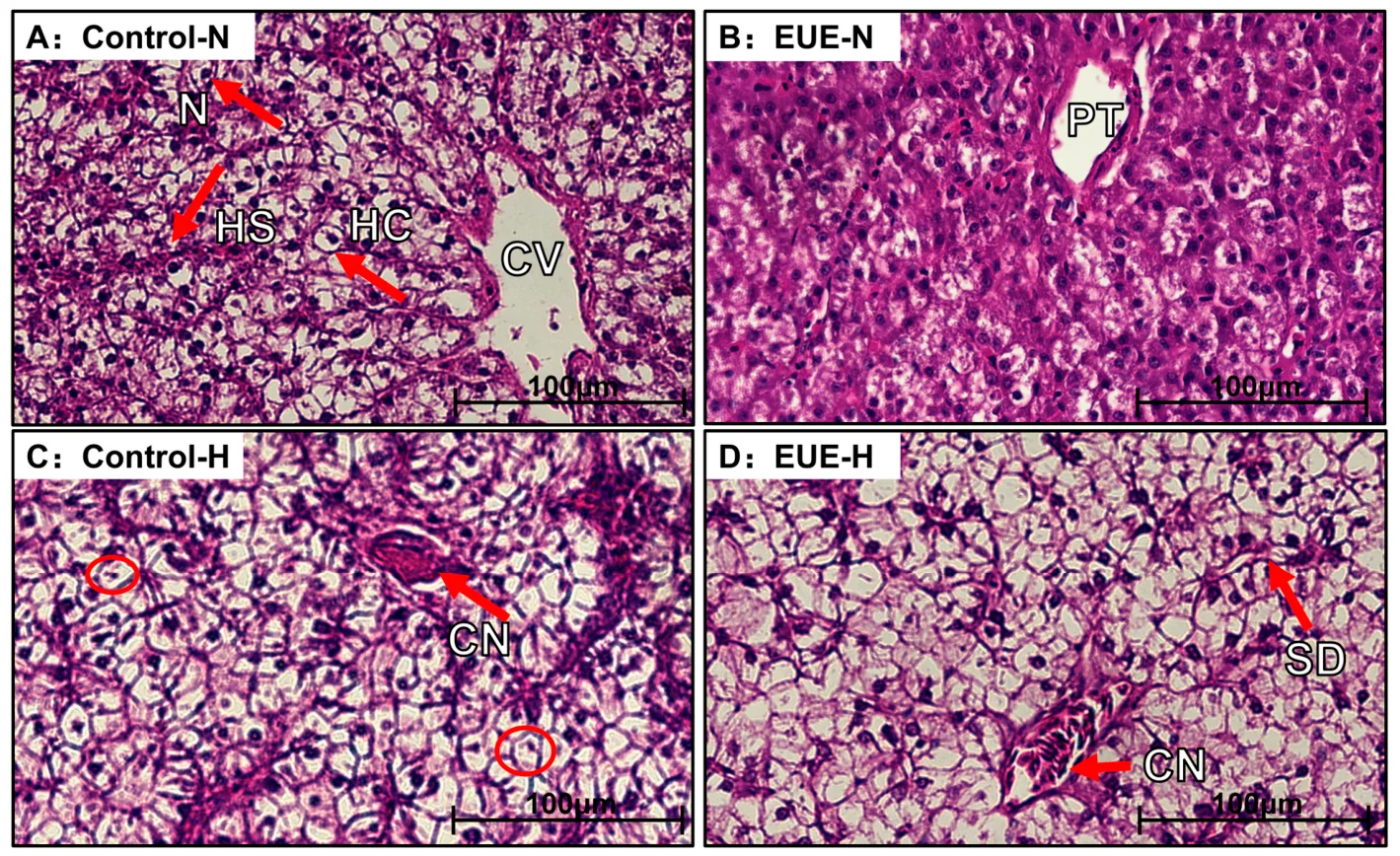
Histopathological observation in liver of largemouth bass. HE-staining (**A**–**D**) ×200. Scale bar = 100 μm. (**A**) Control-N group, (**B**) EUE-N group, (**C**) Control-H group, and (**D**) EUE-H group. (CV: central veins; HC: hepatocyte; N: nucleus; HS: hepatic sinusoid; PT: portal tract; CN: marked vascular congestion; SD: sinusoidal dilatation); malformed nuclei (circle).

The HE-staining results showed that the liver tissue in the Control-N group showed normal distribution of the hepatic sinusoid (Figure 5A), while showing a less orderly arrangement of the hepatocytes and displacement of the nuclei. In the EUE-N group, the liver tissue presented as a healthy phenotype, as evidenced by the orderly arrangement of hepatocytes, uniform nuclear size, and their central position within the cells (Figure 5B). However, when fish were subjected to hypoxic stress, the liver tissue in the Control-H group showed congestion of the portal tract, edema of the hepatic sinusoid, extremely disorganized arrangement of hepatocytes, and severe malformation of the nuclei (Figure 5C). Although there was sinusoidal dilatation of the hepatic sinusoid in the EUE-H group, the phenomenon of marked vascular congestion and hepatic sinusoidal edema was significantly reduced (Figure 5D).

### 3.6. Effects of EUE Under Hypoxic Stress on Antioxidant Parameter of Liver Tissue

The antioxidant parameter in the liver tissue of largemouth bass is shown in Table 3. It is obvious that the content of MDA was apparently increased by hypoxic stress, but the EUE-H group was significantly higher than that in the other groups. On the other hand, the activities of SOD, CAT and GSH-Px in the EUE-H group were significantly lower than the EUE-N group; meanwhile, their activities in the EUE-H group were markedly higher than that in the Control-H group. In addition, T-AOC had the same trend as antioxidant enzymes.

### 3.7. Effects of EUE Under Hypoxic Stress on the Expression of Hif-1α Gene and Nrf2/keap1 Pathway-Related Genes in Liver Tissue

**Figure 6:**
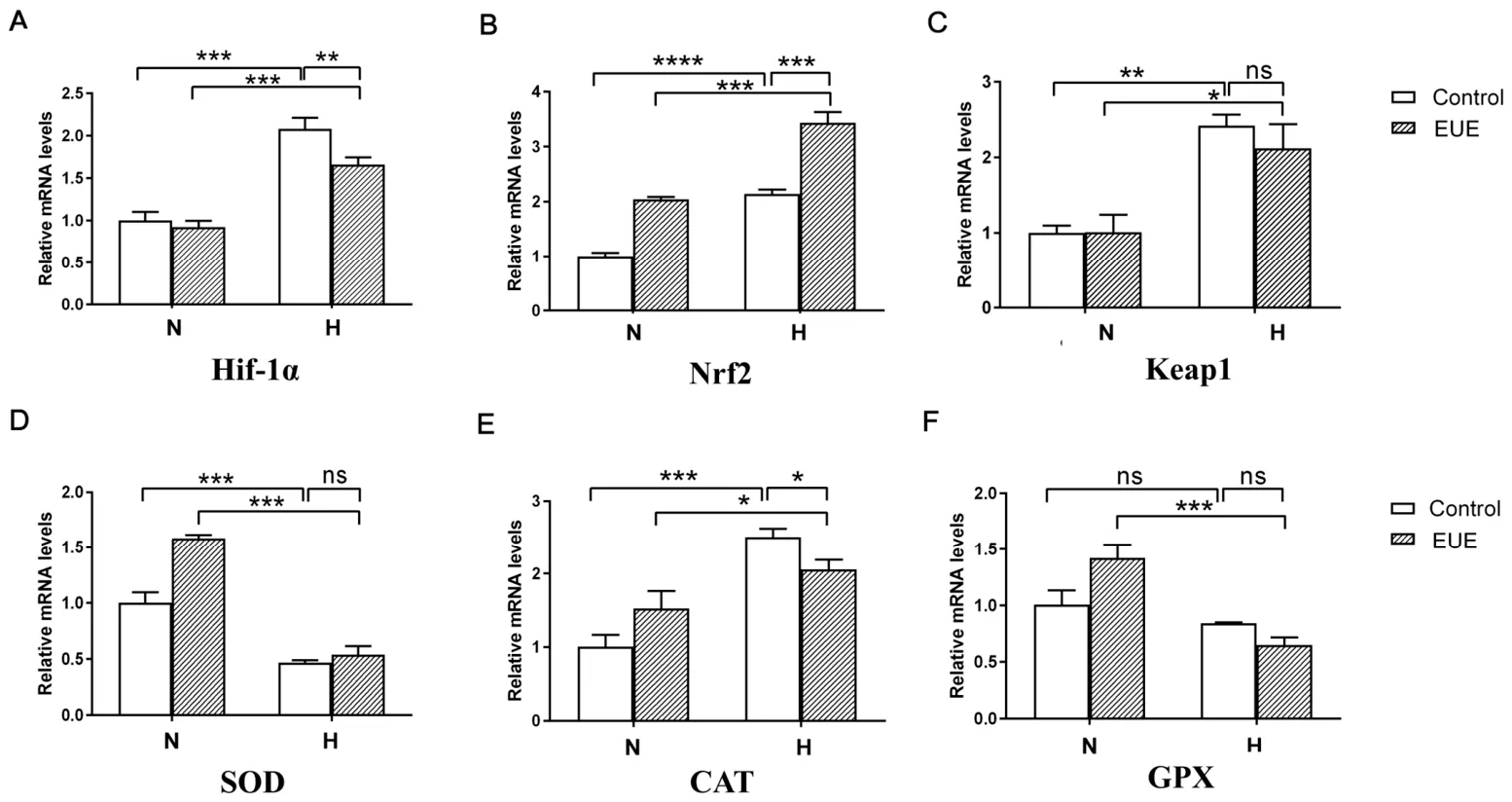
Changes in gene expression levels related to *Nrf2/Keap1* signaling pathway in liver tissue of largemouth bass in normal and hypoxic groups after feeding with EUE. N, normoxia; H, hypoxia. (**A**) *Hif-1α*; (**B**) *Nrf2*; (**C**) *Keap1*; (**D**) *SOD*; (**E**) *CAT*; (**F**) *GPX*. ns indicates no significant difference (*p* > 0.05); the *, **, *** or **** at the top of the bar chart indicates statistically significant; * shows significant difference (*p* < 0.05); **, *** or **** indicate extremely significant difference (*p* < 0.01, *p* < 0.005 or *p* < 0.001). Data are presented as mean ± SD (n = 3, two—way analysis of variance).

Based on Figure 6, it is observed that the expression of *Hif-1α* in Control-H and EUE-H groups was significantly higher than those in Control-N and EUE-N groups (*p* < 0.005, Figure 6A). However, the expression of *Hif-1α* in the EUE-H group was markedly lower than the Control-H group (*p* < 0.01). On the other hand, we examined the expression of *Nrf2*/*keap1* pathway-related genes in liver tissue. Following hypoxic stress, *Nrf2* mRNA expression levels were significantly up-regulated in both the Control-H and EUE-H groups compared to the Control-N and EUE-N groups. (*p* < 0.001, *p* < 0.005, Figure 6B); the mRNA level of *Nrf2* in the EUE-H group was significantly higher (by 1.61-fold) than the Control-H group (*p* < 0.005). Similarly to the expression pattern of *Nrf2*, *Keap1* was also significantly elevated in response to hypoxic stress (Figure 6C). However, EUE supplementation only slightly reduced the hypoxia-induced up-regulation of *Keap1*, as the EUE-H group showed no statistically significant difference from the Control-H group (*p* > 0.05). Regarding antioxidant enzyme genes, the mRNA levels of *SOD* and *GPX* in the EUE-H group exhibited a decreasing trend compared to the Control-H group following hypoxic stress (*p* < 0.005) (Figure 6D,E). Instead of *SOD* and *GPX*, hypoxic stress could noticeably up-regulate the expression of *CAT* in liver tissue relative to the Control-N group and Control-H group (*p* < 0.005, Figure 6F), and the *CAT* expression in the Control-H group was substantially lower than the EUE-H group (*p* < 0.05).

### 3.8. Effects of EUE Under Hypoxic Stress on the Expression of TOR Pathway-Related Genes in Liver Tissue

**Figure 7:**
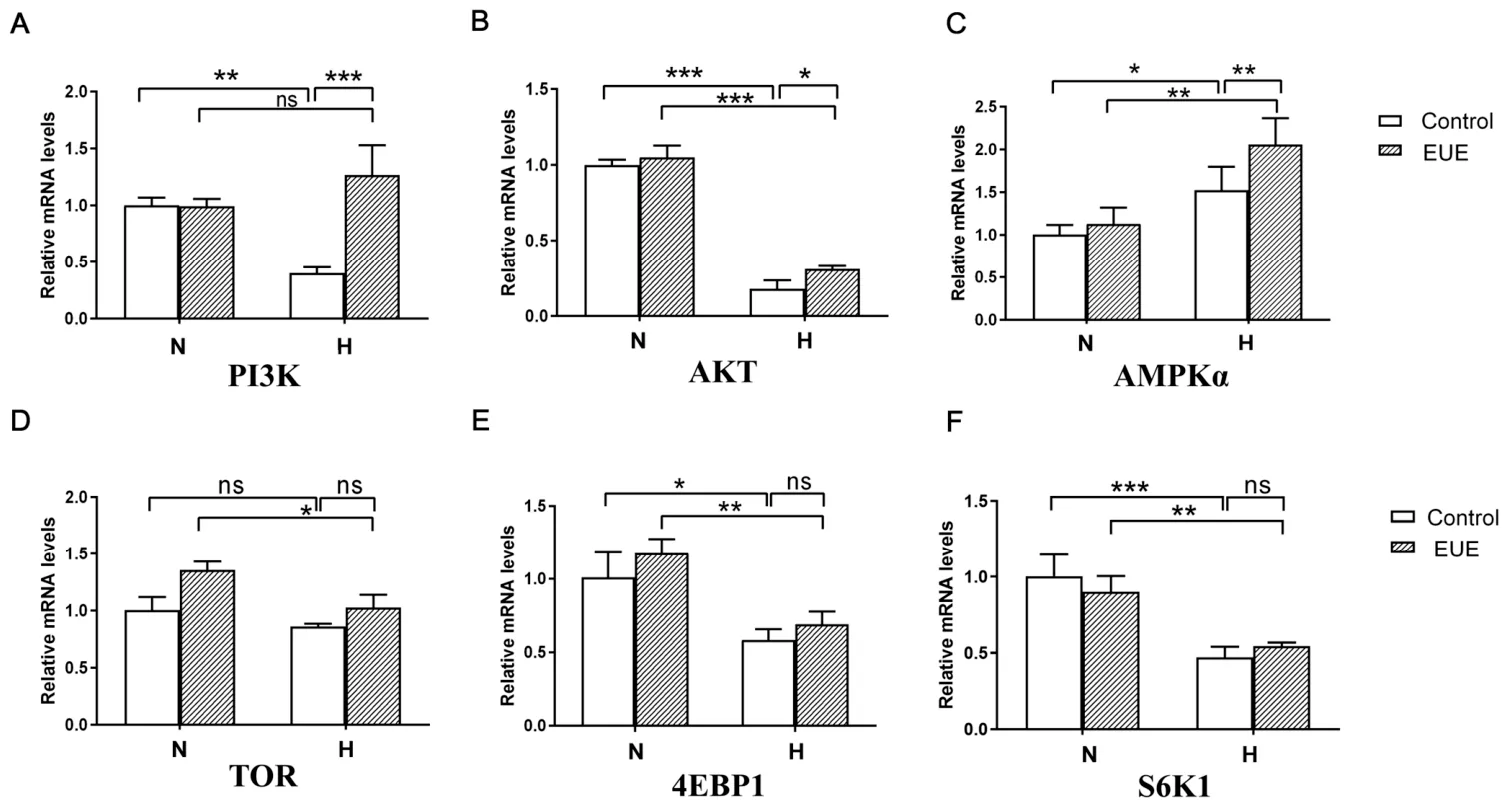
Changes in gene expression levels related to *TOR* signaling pathway in liver tissue of largemouth bass in normal and hypoxic groups after feeding with EUE. N, normoxia; H, hypoxia. (**A**) *PI3K*; (**B**) *AKT*; (**C**) *AMPKα*; (**D**) *TOR*; (**E**) *4EBP1*; (**F**) *S6K1*. ns indicates no significant difference (*p* > 0.05); the *, **, *** at the top of the bar chart indicates statistically significant; * shows significant difference (*p* < 0.05); **, *** indicate extremely significant difference (*p* < 0.01, *p* < 0.005). Data are presented as mean ± SD (n = 3, two-way analysis of variance).

As presented in Figure 7, it is remarkable that when EUE was added to the diet of largemouth bass, the expression of *PI3K* showed no obvious change after hypoxic stress (*p* > 0.05, Figure 7A), while it was considerably down-regulated in the Control-H group and highly markedly lower than the EUE-H group (*p* < 0.05, *p* < 0.005). After hypoxia, the expression of the *AKT* was strongly significantly diminished compared to the normoxia groups (*p* < 0.005, 5.56-fold the Control-N group vs. the Control-H group, 3.89-fold the EUE-N group vs. the EUE-H group, Figure 7B). Moreover, regardless of EUE supplementation, *AMPKα* mRNA levels were significantly elevated following hypoxic stress. (*p* < 0.05, *p* < 0.01, Figure 7C); the *AMPKα* expression level of EUE-H group reached the highest value among all groups. Furthermore, EUE added to the diet could considerably down-regulate the expression of *TOR* in response hypoxic stress (*p* < 0.05, Figure 7D), whereas there was no significant change between the Control-N and Control-H groups (*p* > 0.05). On the other hand, the expression patterns of *TOR* downstream genes, *4EBP1* and *S6K1*, were found to be similar (Figure 7E,F). This is reflected in the down-regulation of the expression of *4EBP1* and *S6K1* under hypoxic stress. Meanwhile, there was no substantial difference in the *4EBP1* and *S6K1* mRNA levels between the Control-H and EUE-H groups (*p* > 0.05) in liver tissue.

### 3.9. Effects of EUE Under Hypoxic Stress on the Expression of ER Stress-Related Genes in Liver Tissue

**Figure 8:**
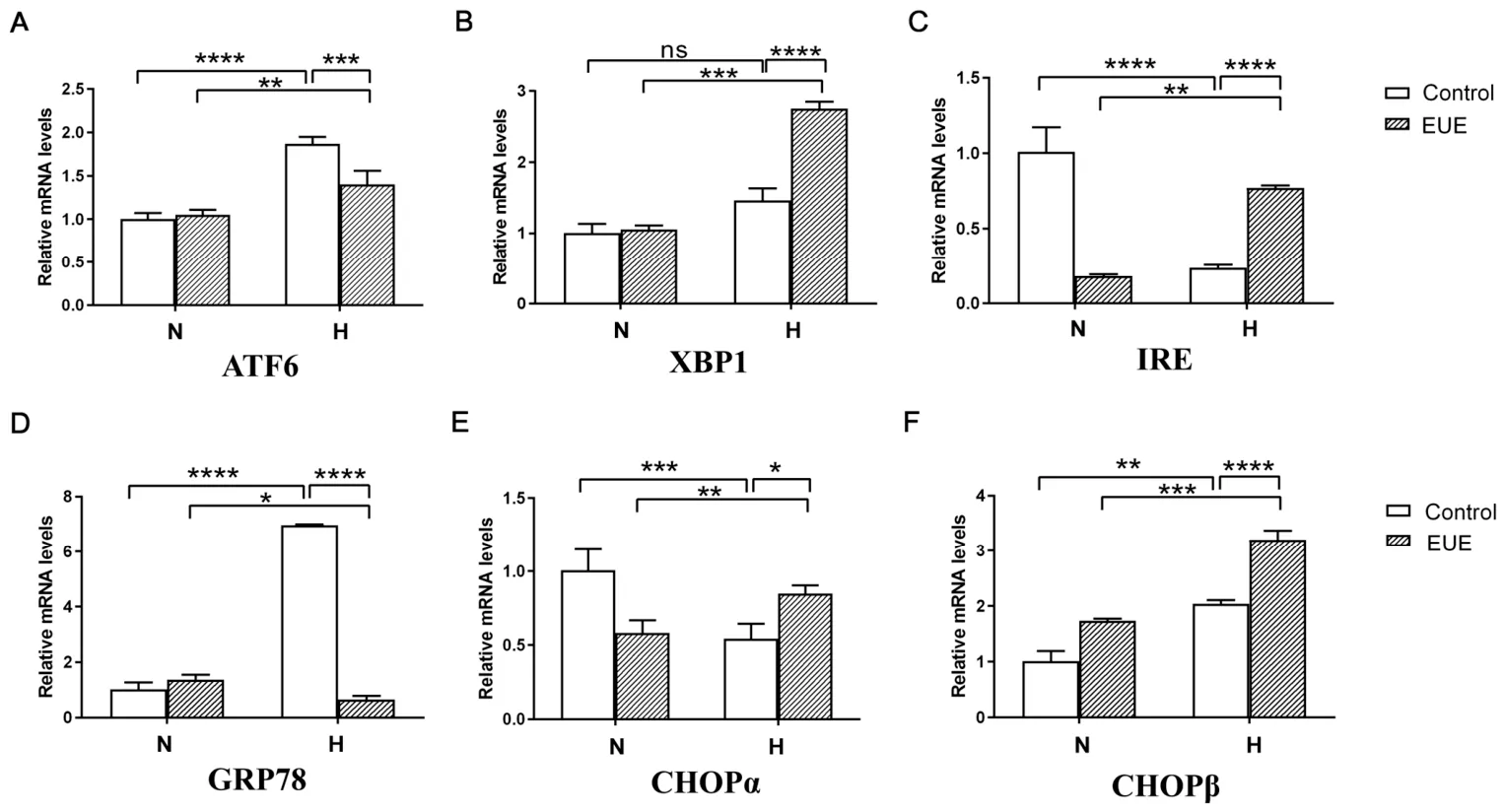
Changes in gene expression levels related to endoplasmic reticulum stress in liver tissue of largemouth bass in normal and hypoxic groups after feeding with EUE. N, normoxia; H, hypoxia. (**A**) *ATF6*; (**B**) *XBP1*; (**C**) *IRE*; (**D**) *GRP78*; (**E**) *CHOPα*; (**F**) *CHOPβ*. ns indicates no significant difference (*p* > 0.05); the *, **, *** or **** at the top of the bar chart indicates statistically significant; * shows significant difference (*p* < 0.05); **, *** or **** indicate extremely significant difference (*p* < 0.01, *p* < 0.005 or *p* < 0.001). Data are presented as mean ± SD (n = 3, two—way analysis of variance).

According to the data in Figure 8, the mRNA expression of *GRP78* in liver tissue of the Control-H group reached the highest expression among all groups (Figure 8D). Nevertheless, after feeding with EUE, there was a significant decrease in the expression of *GRP78* under hypoxic conditions (*p* < 0.05). Additionally, *ATF6* and *XBP1* were maintained at relatively low levels in normoxia conditions, but their expression could be up-regulated upon exposure to hypoxic stress (Figure 8A,B). The expression of *ATF6* in the EUE-H group was extremely sharply lower than the Control-H group (*p* < 0.005), whereas the *XBP1* expression level showed the opposite trend between the two groups. It is noteworthy that the expression of *IRE* in the Control-H group was extremely dramatically lower than the Control-N group (*p* < 0.001, Figure 8C). However, after treatment with EUE, the *IRE* expression level was elevated under hypoxic stress, with its expression in the EUE-H group being higher than the Control-H group (*p* < 0.001). In addition, hypoxic stress significantly suppressed *CHOPα* expression in the control diet conditions, as evidenced by the comparison between the Control-N and Control-H groups (*p* < 0.005; Figure 8E). However, EUE supplementation reversed this trend, with *CHOPα* expression in the EUE-H group being significantly higher than that in the Control-H group (*p* < 0.05). On the other hand, hypoxic stress could significantly up-regulate the mRNA levels of *CHOPβ* in the Control and EUE groups (*p* < 0.01, *p* < 0.001, Figure 8F). Ultimately, it is interesting that the *CHOPβ* expression level in the EUE-H group was higher than the Control-H group (*p* < 0.001).

### 3.10. Effects of EUE Under Hypoxic Stress on the Expression of Apoptosis-Related Genes in Liver Tissue

**Figure 9:**
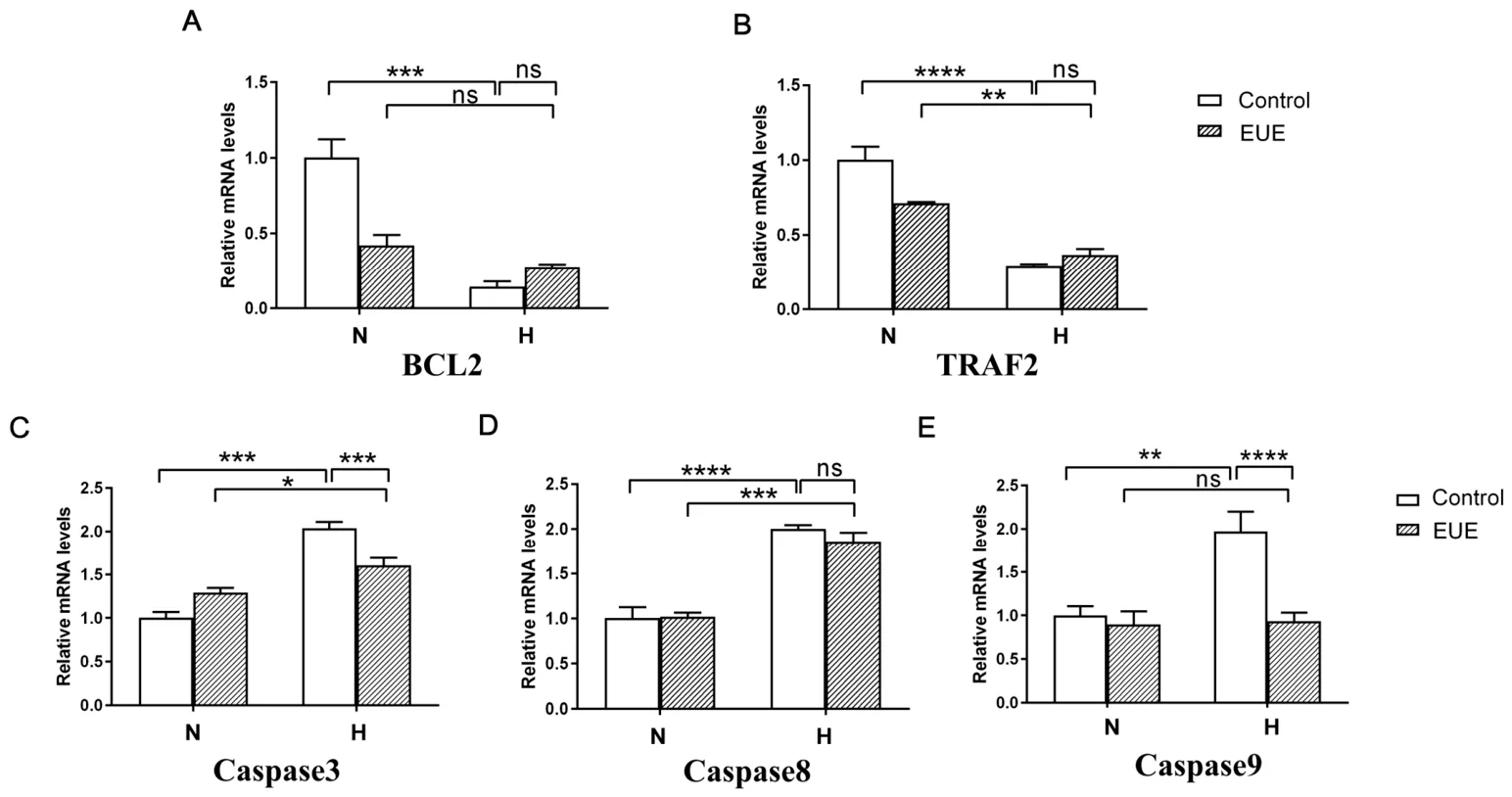
The difference in gene expression levels of apoptosis-related genes in liver tissue of largemouth bass in normal and hypoxic groups after feeding with EUE. N, normoxia; H, hypoxia. (**A**) *BCL2*; (**B**) *TRAF2*; (**C**) *Caspase3*; (**D**) *Caspase8*; (**E**) *Caspase9.* ns indicates no significant difference (*p* > 0.05); the *, **, *** or **** at the top of the bar chart indicates statistically significant; * shows significant difference (*p* < 0.05); **, *** or **** indicate extremely significant difference (*p* < 0.01, *p* < 0.005 or *p* < 0.001). Data are presented as mean ± SD (n = 3, two-way analysis of variance).

In accordance with the results in Figure 9, hypoxic stress could remarkably down-regulate the expression of the anti-apoptotic gene *BCL2* in the Control groups (*p* < 0.005, Figure 9A). Moreover, under both control and EUE dietary conditions, the mRNA level of *TRAF2* was significantly reduced after hypoxic stress (*p* < 0.001, *p* < 0.01, Figure 9B). Under hypoxic conditions, the addition of EUE did not significantly affect the expression of *BCL2* and *TRAF2*. (*p* > 0.05). In order to observe the effects of EUE and hypoxia on the apoptosis of largemouth bass, the expression of some genes in the caspase family was also detected. Hypoxic stress significantly increased *caspase3* expression in the liver tissue (Control-H vs. Control-N, *p* < 0.005, EUE-H vs. EUE-N, *p* < 0.05, Figure 9C). However, the magnitude of this increase was markedly lower in the EUE-H group than in the Control-H group. Moreover, under hypoxic conditions, *caspase3* expression in the EUE-H group was significantly lower than that in the Control-H group (*p* < 0.005). Likewise, the *caspase8* mRNA level showed a similar trend to *caspase3* (Figure 9D), but there was no significant difference between the EUE-H group and Control-H group (*p* > 0.05). On the other hand, by comparing the expression levels of *Caspase9* between Control-N and Control-H groups, it was found that hypoxic stress significantly increased *Caspase9* expression (*p* < 0.01, Figure 9E). However, there was no significant difference in *Caspase9* expression between EUE-N and EUE-H groups (*p* > 0.05). At the same time, it is worth noting that under the same hypoxic conditions, the expression level of *Caspase9* in the EUE-H group was significantly lower than that in the Control-H group. (*p* < 0.001).

## 4. Discussion

### 4.1. EUE Alleviates Deterioration of Plasma and Hematological Parameter Caused by Hypoxic Stress

For most fish species, hypoxia is thought to cause physiological disturbances and have a negative influence on behavior, physiology, immunity, growth, and reproduction [60]. As a result, fish have evolved and employed complex physiological regulatory strategies to cope with hypoxic stress and attempt to maintain homeostasis [61]. Actually, blood is an important site for energy metabolism, physiological regulation and defense of the body [62]. On one hand, the total protein content of plasma can reflect the nutritional status and protein level of fishes; on the other hand, it indirectly reflects the non-specific immune level of the body [63]. In this study, the plasma total protein of largemouth bass decreased significantly under hypoxic stress. It has been reported that the plasma total protein levels of sea bass (*L. maculatus*) and cobia (*Rachycentron canadum*) were apparently reduced under hypoxic stress, which is consistent with the results of this study [64,65]. These results suggest that the immune system of fish was inhibited during hypoxic stress. In addition, previous studies have shown that plasma GLU levels increase significantly during hypoxic stress due to the increased plasma content of cortisol, a product of the hypothalamus–pituitary–interrenal axis, which promotes liver gluconeogenesis and glycogenolysis, thereby increasing blood GLU levels [66,67]. Studies of Nile tilapia (*Oreochromis niloticus*) and spotted wolffish (*Anarhichas minor*) have shown that plasma GLU levels increase significantly during hypoxic stress [68,69]. However, in this study, the plasma GLU level of largemouth bass decreased obviously during acute hypoxic stress with the plasma GLU level in the EUE-H group being evidently higher than those in the Control-H group. This indicates that EUE may respond to lactate accumulation caused by hypoxia through the hepatic gluconeogenesis pathway, and can quickly restore the levels of anaerobic glycolysis products in the blood during hypoxia, ensuring the stable survival of the organism in an oxygen-deficient environment. GLU, TG and CHOL are commonly used as indicators to assess the nutritional status of fish [70]. Hypoxia can lead to reduced plasma CHOL levels in fish by stimulating phospholipid hydrolysis and inhibiting the β-oxidation of epinephrine [71]. Therefore, the parameters of TP, GLU, TG and CHOL in plasma, which are susceptible to stress conditions, are often used to evaluate the stress response and adaptability of fish to environmental changes. Studies in cobia showed that TG and CHOL decreased after hypoxic stress (DO = 2.64 ± 0.25 mg/L, 3 h) [64]. In contrast, the plasma TG content of turbot (*Scophthalmus maximus* L.) was significantly increased under acute hypoxic stress, while CHOL remained unchanged during hypoxic stress (DO = 1.30 ± 0.16 mg/L, 8 h) [72]. In this study, the plasma TG content of largemouth bass decreased notably after hypoxic stress with the plasma TG level in the EUE-H group being evidently higher than that in Control-H group, while CHOL content had no statistically significant change. From these results, it is clear that the differences in TG and CHOL levels may be due to fish-specific responses or the different physiological states of the fish. The up-regulation of TG level in the EUE-H group suggests that EUE may promote lipid catabolism during hypoxic stress.

Furthermore, hematological characteristics of fishes such as total blood cell count (TBCC), red cell count (RBC) and white cell count (WBC) are often used to assess the oxygen-carrying capacity of fish to indicate the physiological status and health status of fish under different environmental stressors [73]. In our previous study in largemouth bass, it was observed that both the levels of WBC and RBC have significantly decreased after acute hypoxic stress (DO = 4.33 mg/L and DO = 3.25 mg/L) [23]. However, the number of TBCC of largemouth bass in the EUE-H group was visibly higher than that in the Control-H group; instead, the number of macrophages in EUE-H group was slightly decreased. The results lead to the indication that EUE was beneficial to promote the TBCC, which helps to improve the ability of fish to obtain and transport more oxygen, thus alleviating the hypoxia and stress state of the body.

### 4.2. EUE Improves Liver Tissue Damage Induced by Hypoxic Stress

Normally, ALT and AST in the blood are involved in protein metabolism and energy conversion in fish [74]. It has been reported that when fish liver is exposed to environmental stressors, the damage of liver tissue increases the permeability of the liver cell membrane, which increases the release of enzymes such as ALT and AST into the blood [75]. Subsequently, the released ALT and AST then participate in stress-induced gluconeogenesis [76]. This study found that plasma ALT and AST levels were markedly increased after exposure to the hypoxic environment, while plasma ALT and AST levels in the EUE-H group were significantly lower than those in the Control-H group. Previous studies also showed similar results whereby the serum ALT and AST levels of cobia and sea bass were remarkably increased after hypoxic stress [64,65]. These results indicate that hypoxia leads to tissue damage of the liver, while the supplementation of EUE to the diet may alleviate the liver damage caused by hypoxia.

Correspondingly, the results of HE-staining micrographs of liver tissue sections in this study also reflected the same conclusion. Liver, the primary organ of fish metabolism, plays a crucial role in metabolism, growth, detoxification and immunity, which is one of the most sensitive organs to various environmental stressors such as hypoxia [77,78]. Yet, the liver is conductive to the adaptation of aquatic organisms to hypoxia [79]. Additionally, the study of grass carp found that some hepatocyte necrosis, vacuolation and dilatation of hepatic blood sinuses occurred in the liver after hypoxic stress [80]. In line with previous studies, this study displayed that the largemouth bass in the Control-H group was subjected to hypoxia stress with disordered hepatocyte arrangement, portal vein congestion, partial hepatocyte edema and nuclear deformity. However, we were surprised to find that most of the largemouth bass in the EUE-H group still arranged orderly after hypoxic stress, the degree of venous congestion was relatively light and the number of hepatocytes with nuclear deformity also showed a downward trend. From these results, it is clear that hypoxic stress can lead to different degrees of liver damage in different species. Interestingly, dietary supplementation of EUE can ameliorate the liver tissue damage induced by hypoxic stress in largemouth bass.

### 4.3. EUE Increased the Activity of Antioxidant Enzymes and Activated the Nrf2/Keap1 Signaling Pathway of Liver to Enhance Antioxidant Capacity, Reduce the ROS Content and Oxidative Damage

It is universally known that hypoxic stress can disrupt the normal physiological functions of fish for the reason that it can cause mitochondrial dysfunction in fish and accelerate the production of ROS [81]. Moreover, the overproduction of ROS may lead to lipid peroxidation, protein carbonylation and DNA damage, which cause oxidative stress to the body [82]. Normally, Hif-1α degrades rapidly under normoxia conditions and is constitutively expressed encountering hypoxic stress, because the *Hif-1α* gene is up-regulated and triggered in response to low oxygen levels and the mitochondrial overproduction of ROS [83]. Quite a few experimental studies have exhibited that Hif-1α is a vital transcription factor that regulates a variety of genes during hypoxia in mammals and bony fish species, which can activate the expression of target genes involved in oxygen transport, antioxidant enzymes and energy homeostasis [84]. The results of our study demonstrated that ROS levels in cells and Hif-1α protein expression in liver were dramatically up-regulated after acute hypoxic stimulation. However, it is a remarkable fact that the supplementation of EUE in the diet reduced the production of ROS and induction of *Hif-1α* mRNA level in liver under hypoxic stress, indicating that the response of EUE-fed largemouth bass to hypoxic stress was decreased. For thousands of years, natural medicine has been widely used in the prevention and treatment of various diseases because of its considerable effectiveness and high safety [85]. As a Chinese herbal medicine, EUE has been proved to inhibit inflammation and antioxidant damage in channel catfish and large yellow croaker (*Larimichthys crocea*) [86]. In addition, MDA is the product of lipid peroxidation in the process of oxidative stress, which can reflect the oxidation state of the body [87]. It has been identified that MDA levels of shrimp and largemouth bass were significantly up-regulated under hypoxic stress [88,89]. However, most fish can remove excess free radicals and reduce oxidative damage through non-enzyme system and antioxidant enzyme system including SOD, CAT and GSH-Px [90]. In this study, compared to the normoxia group, the activities of SOD, CAT and GSH-Px in the liver of largemouth bass in the hypoxia group were down-regulated and the T-AOC was decreased, while the activities of SOD, CAT and GSH-Px and T-AOC in the EUE-H group were significantly higher than those in the Control-H group. Similarly, the activities of CAT, SOD, GSH-Px and T-AOC in liver of gibel carp were decreased after acute hypoxic stimulation (DO = 0.08 ± 0.02 mg/L, 3 h) [4]. However, it was found in the study of blunt snout bream (*Megalobrama amblycephala*) that SOD and GSH-Px activities in liver and the activities of SOD and CAT in intestine were remarkably increased after hypoxia [91]. Therefore, the response of antioxidant enzymes and antioxidant capacity may vary by species and tissue. Consequently, these results imply that hypoxia induced excessive accumulation of ROS and MDA, which may lead to serious oxidative damage in the body, while EUE can increase the activities of SOD, CAT, GSH-Px and enhance T-AOC.

Nrf2, as an important transcription factor, binds to Keap1 in basal conditions, which regulates its ubiquitination and proteasome degradation [92]. When exposed to environmental stressors such as hypoxia, Nrf2 dissociates from Keap1; thus, the free Nrf2 accumulates and translocates to the nucleus, which binds to antioxidant response elements (AREs) and regulates the expression of multiple genes involved in antioxidant responses, such as *SOD*, *CAT*, and *GPx* [93]. Interestingly, some Nrf2 activators, such as sulforaphane, can effectively reduce lipid peroxidation levels and inflammatory responses by stimulating Nrf2 nuclear translocation to promote a series of endogenous cytoprotective gene expression [94]. Furthermore, it was found that EUE can also protect cells via up-regulating *Nrf2* gene expression and reducing oxidative stress [95]. SOD, CAT and GSH-Px are important antioxidant enzymes that can remove excessive free radicals and ROS and prevent lipid peroxidation in the body [96]. At present, there is evidence that flavonoids in EUE bind well with proteins related to lipid metabolism, which can inhibit oxidative stress and protect the liver [97]. The results of this study showed that the mRNA levels of *Nrf2*, *Keap1* and *CAT* were prominently increased after hypoxic stress; instead, the expression levels of *SOD* and *GPx* genes were significantly down-regulated. Together, the present findings confirm that hypoxia can activate the Nrf2/Keap1 signaling pathway, and the decrease in *SOD* and *GPx* expression levels may be the compensation for the up-regulation of *CAT* gene expression. In addition, our study demonstrated that the expression level of *Nrf2* in the liver of largemouth bass in EUE-H group was markedly higher than that in the Control-H group, while the mRNA level of *CAT* was significantly lower than that in the Control-H group. These results suggest that dietary EUE can promote the expression of *Nrf2* and *SOD*. In general, the present study confirmed the findings that EUE can act as an Nrf2 activator, activate the Nrf2/Keap1 signaling pathway and relieve the oxidative stress state of largemouth bass.

### 4.4. EUE Inhibited Apoptosis Induced by Hypoxia via Activating PI3K/AKT/AMPKα Signaling Pathway

Endothelial cells (ECs) are the first layer exposed to changes in oxygen levels and are sensitive to changes in oxygen concentration [98]. NO is a crucial bioactive molecule produced by ECs, which has a considerable effect on vasodilation and contraction, anti-inflammatory and so on [99]. Additionally, NO has been reported to exhibit different concentration-dependent effects: on one hand, it has a vasodilating effect at low levels, on the other hand, it can induce apoptosis through a variety of mechanisms, including inhibiting *Bcl-2* expression and aggravating oxidative stress at high levels [100,101]. In this study, the production of NO in the blood cells of largemouth bass was significantly up-regulated after hypoxic stress. Interestingly, the content of NO in the blood cells of the EUE-H group was remarkably lower than that of the Control-H group, suggesting that EUE may play a positive role in alleviating the oxidative stress of largemouth bass. Apoptosis is an extremely complex process involving multiple signaling pathways, including PI3K, AMPKα and TOR pathways [102]. PI3K controls a variety of cellular processes, such as growth, survival, metabolism, autophagy and apoptosis with AKT acting as the main effecting factor of PI3K [103,104]. As reported in the study of goldfish, exposure to low oxygen is accompanied by the activation of PI3K/AKT signaling [105]. Moreover, TOR is a major negative regulator of autophagy and apoptosis, which is mainly involved in nutrient metabolism and oxidative stress [106]. Surprisingly, AMPKα has been identified as an essential factor in AKT phosphorylation and activation under various stressors, such as hypoxia [107]. The discovery of the AMPK-activated AKT pathway not only provides insight into how AMPK plays a protective and survival role under different stress conditions, but it may also extend to other stress conditions, such as ER stress and DNA damage [107]. In addition, AMPKα, an energy-sensitive kinase and a negative regulator of TOR, plays a vital role in responding to various stressors and inducing autophagy [108]. Study in gibel carp has proved that acute hypoxia mediates the AMPKα/TOR pathway by activating AMPKα and inhibiting TOR protein expression [109]. In the study of mice, hypoxia primarily activated the AMPK/ TOR signaling pathway [110]. Furthermore, many studies have found that plant extracts such as salidroside and emodin can act as AMPKα activators and have a positive influence on protecting cells under hypoxic stress [111,112]. At present, this study demonstrated that the mRNA levels of *PI3K*, *AKT* and *AMPKα* genes in the liver of EUE-fed largemouth bass after acute hypoxic stress were notably up-regulated compared to those of fish-fed control diet. These results suggest that EUE probably acted as the activator of *AMPKα* to inhibit *TOR* expression by mediating the PI3K/AKT/AMPKα signaling pathway, thereby inducing autophagy and inhibiting apoptosis to protect hepatocytes under hypoxic conditions. However, further studies are needed to discover the specific molecular mechanism of how EUE mediates its cytoprotective effect by regulating autophagy.

### 4.5. EUE Alleviates ER Stress and Inhibits Apoptosis by Activating the UPR

The ER is a crucial organelle responsible for protein synthesis, modification and Ca^2+^ storage [113,114]. However, ER is sensitive to hypoxia, whereby oxidative stress caused by hypoxia can disrupt the homeostasis of ER [115]. Many recent studies have shown that exposure to hypoxic environments leads to extensive protein modification and the accumulation of unfolded proteins in the ER, causing ER stress and the disruption of Ca^2+^ homeostasis in the intracellular environment and even apoptosis [116,117]. In addition, for Ca^2+^, a highly flexible second messenger, other studies have found that it controls key reactions in eukaryotic cells, which is also involved in responses to cold, hypoxia and other environmental stresses [118,119]. In study of largemouth bass, hypoxia can lead to a significant increase in Ca^2+^ levels in the cytoplasm [120]. Consistent with previous studies, this study displayed that the concentration of Ca^2+^ in the blood cells of largemouth bass fed the control diet was evidently up-regulated after hypoxic stress, while the level of Ca^2+^ in the blood cells of EUE-fed fish did not change significantly after hypoxic stress, suggesting that EUE could inhibit ER stress induced by hypoxia. According to the expression of ER stress-related genes, when ER stress occurs, signaling pathways such as ATF6 and IRE-XBP1 are activated to trigger UPR [121]. UPR is an adaptive and protective mechanism under stress states that reduces the rate of protein synthesis, improves protein folding capacity and helps cells degrade misfolded or unfolded proteins through a variety of pathways [122]. It was found that exposure to hypoxia significantly up-regulated the expression levels of *ATF6*, *XBP1* and *IRE* genes in the liver of yellow catfish (*Pelteobagrus fulvidraco*) [123]. Additionally, GRP78 is an ER chaperone that regulates ER stress via combining IRE and ATF6 [124]. Previous study has evidence that hypoxia exposure can promote the binding of GRP78 to IRE/ATF6, which reduced the unfolded protein response, activated ER overload response and eventually induced cell death [125]. In this study, it is a remarkable fact that the mRNA levels of *GRP78* and *ATF6* in the liver of largemouth bass supplemented with EUE were notably decreased after hypoxic stress was compared to the Control-H group, while the expression of *XBP1* and *IRE* were significantly up-regulated. Consequently, the present findings indicate that EUE may increase UPR by inhibiting the expression of *GRP78* and promoting the expression of *XBP1* and *IRE*, relieving ER stress and inhibiting apoptosis, while the decreased expression of *ATF6* may be a compensatory effect.

Apoptosis is an important defense mechanism of the body and plays an important role in maintaining the homeostasis of the body; however, excessive apoptosis can cause damage to normal tissues and affect their regular functions [126]. Recent studies have found that excessive environmental stress can induce cell apoptosis. Furthermore, due to the special respiratory pattern and habitat of fish, they are more susceptible to hypoxia, which induce oxidative stress and lead to apoptosis [127]. Hypoxia-induced apoptosis of fish cells has been confirmed in several studies; for example, in the studies of *Hypophthalmichthys molitrix* and darkbarbel catfish (*Pelteobagrus vachelli*), hypoxic stress significantly promotes the apoptosis of liver cells [128,129]. Similar results were obtained in this study that the apoptosis rate of largemouth bass blood cells was significantly up-regulated after acute hypoxic stress. Additionally, it has been proved that mitochondria-mediated apoptosis is one of the main ways of hypoxia-induced apoptosis [130]. Specifically, mitochondria-mediated apoptosis is controlled by the BCL2 family. When apoptosis occurs, *BCL2* expression is down-regulated; afterwards, it promotes the release of cytochrome c and activates *Caspase 9* to form apoptotic corpuscles, which ultimately increases the expression of *Caspase 3* and leads to cell apoptosis [131]. Previous studies have displayed that hypoxic stress significantly stimulated the expression of *Caspase 3* and down-regulated the expression of *BCL2* in *Zebrafish* and *Pelteobagrus vachelli* [132]. In the present study, the findings are directly in line with previous findings that the expression levels of *Caspase3* and *Caspase9* in EUE-H group were prominently decreased compared to the Control-H group. In general, these results suggest that EUE can protect the liver by inhibiting apoptosis induced by hypoxia.

## 5. Conclusions

In conclusion, acute hypoxia induced oxidative injury, liver tissue lesion and ER stress, which were associated with the apoptosis of largemouth bass cells. Our current study suggested that (1) EUE supplementation may be associated with the activation of the Nrf2/Keap-1 pathway, and enhance the antioxidant capacity of largemouth bass to alleviate the liver tissue damage caused by acute hypoxia; (2) EUE may promote UPR to relieve ER stress by activating the expression of *XBP1* and *IRE* and down-regulating the mRNA level of *GRP78*; (3) EUE inhibits apoptosis under acute hypoxic stress by down-regulating the expression levels of *Caspase3* and *Caspase9*. Consequently, these findings suggest that EUE may reduce oxidative damage and cell apoptosis through mechanisms involving Nrf2/Keap1-mediated ER stress and alleviating the detrimental effects of acute hypoxic stress on largemouth bass. This study may provide some theoretical reference for promoting the green and healthy development of largemouth bass farming.

## Figures and Tables

**Table 1:** Ingredients and composition of the experimental diets (dry matter-based)% [49,50,51].

Ingredients	Control	EUE
Fish meal	45.00	45.00
EUE	0.00	0.10
Soybean meal	16.00	16.00
Soybean protein concentrate	16.60	16.60
α-starch	12.50	12.40
Monocalcium phosphate	1.50	1.50
Fish oil	2.00	2.00
Soybean oil	3.50	3.50
Lecithin	1.00	1.00
L-Ascorbate-2-Monophosphate	0.30	0.30
Betaine	0.50	0.50
Vitamin premix ^1^	0.10	0.10
Mineral premix ^2^	0.50	0.50
Chloride choline	0.50	0.50
Total (g)	100.00	100.00
Nutrient levels ^3^		
Crude protein	49.29	48.96
Crude lipid	9.68	9.12
Moisture	4.09	4.89
Ash	11.32	10.8
Ca	1.76	1.69
P	1.76	1.66

^1^ Vitamin premix (/kg) provides the following: vitamin A, 3,200,000 IU; vitamin B1, 4 g; vitamin B2, 8 g; vitamin B12, 0.016 g; vitamin D, 1,600,000 IU; vitamin E, 16 g; vitamin K, 4 g; niacin, 28 g; inositiol, 40 g; calcium pantothenate, 16 g; biotin, 0.064 g; folic acid, 1.28 g. ^2^ Mineral premix (/kg) provides the following: MgSO_4_·H_2_O, 12 g; Ca(IO_3_)_2_, 9 g; KCl, 36 g; Met-Cu, 1.5 g; ZnSO_4_·H_2_O, 10 g; FeSO_4_·H_2_O, 1 g; Met-Co, 0.25 g; NaSeO_3_, 0.0036 g. ^3^ Measured values.

**Table 2:** The plasma metabolites of largemouth bass in normal and hypoxic groups after feeding with EUE.

Items	Control-N	EUE-N	Control-H	EUE-H
TP/(g/L)	34.67 ± 0.45 ^b^	38.53 ± 0.81 ^a^	34.07 ± 0.57 ^b^	34.83 ± 2.63 ^b^
GLU/(mmol/L)	5.15 ± 0.10 ^a^	4.9 ± 0.06 ^ab^	4.29 ± 0.21 ^c^	4.6 ± 0.21 ^b^
TG/(mmol/L)	9.78 ± 0.16 ^a^	9.26 ± 0.16 ^a^	6.24 ± 0.92 ^c^	8.12 ± 0.32 ^b^
CHOL/(mmol/L)	7.99 ± 0.18 ^a^	7.92 ± 0.77 ^a^	7.63 ± 0.32 ^a^	7.54 ± 0.45 ^a^
ALT/(U/L)	19.93 ± 7.73 ^b^	8.23 ± 1.07 ^c^	31.31 ± 4.16 ^a^	19.96 ± 0.98 ^b^
AST/(U/L)	158 ± 17.35 ^c^	140.5 ± 2.91 ^c^	256.82 ± 19.31 ^a^	204.92 ± 8.51 ^b^

Values with different letters within the same line indicate significant difference (*p* < 0.05). Data are presented as the mean ± SD (n = 3, one-way ANOVA test of variance).

**Table 3:** Changes in antioxidant parameter in liver tissue of largemouth bass in normal and hypoxic groups after feeding with EUE.

Items	Control-N	EUE-N	Control-H	EUE-H
T-AOC/(U/mg)	1.51 ± 0.01 ^c^	2.62 ± 0.03 ^a^	1.32 ± 0.03 ^d^	2.28 ± 0.08 ^b^
SOD/(U/mg prot)	70.05 ± 4.43 ^c^	97.27 ± 0.79 ^a^	53.56 ± 2.66 ^c^	70.44 ± 1.28 ^b^
MDA/(mmol/mg prot)	2.95 ± 0.01 ^c^	3.05 ± 0.04 ^c^	23.24 ± 0.07 ^b^	29.44 ± 0.05 ^a^
CAT/(U/mg prot)	19.98 ± 1.08 ^b^	24.87 ± 2.18 ^a^	12.30 ± 0.89 ^d^	17.26 ± 0.28 ^c^
GSH-Px/(U/mg prot)	62.98 ± 3.16 ^c^	143.28 ± 3.14 ^a^	44.47 ± 1.97 ^d^	83.39 ± 1.52 ^b^

Values with different letters within the same line indicate significant difference (*p* < 0.05). Data are presented as the mean ± SD (n = 3, one-way ANOVA test of variance).

## Data Availability

The original contributions presented in this study are included in the article.

## Supplementary Materials

The following supporting information can be downloaded at: https://www.mdpi.com/article/10.3390/antiox15091207/s1, Table S1. Sequences of primers used for qRT-PCR.
