# Supplementary material for: Eucommia ulmoides Extract Attenuates Oxidative Stress, Endoplasmic Reticulum Stress and Apoptosis Induced by Acute Hypoxia via the Nrf2/Keap1 Pathway in Largemouth Bass (Micropterus salmoides)
Source: Antioxidants (Basel). 2026 Sep 20;15(9):1207. doi: 10.3390/antiox15091207 (PMC13603811; doi:10.3390/antiox15091207)
Supplement: Supplementary file 1 [file antioxidants-15-01207-s001.zip › antioxidants-4524270-supplementary.pdf]

Table S1. Sequences of primers used for qRT-PCR.

Primer	Nucleotide Sequence (5′→3′)	Length	Accession No.
β-actin-F	AAAGGGAAATCGTGCGTGAC	184	XM_038695351.1
β-actin-R	AAGGAAGGCTGGAAGAGGG		
Hif-1α-F	CAGAGGACCTGTTGAATCGTT	178	XM_038729572.1
Hif-1α-R	TTGTAGATGACAGTGGCTTGG		
Nrf2-F	CAGACAGTTCCTTTGCAGGC	116	XM_038720536.1
Nrf2-R	AGGGACAAAAGCTCCATCCA		
Keap1-F	CAGCATTACATGGCCGCATC	86	XM_038713667.1
Keap1-R	CTTCTCTGGGTCGTAAGACTCC		
SOD-F	CCACCAGAGGTCTCACAGCA	158	XM_044189234.1
SOD-R	CCACTGAACCGAAGAAGGACT		
CAT-F	GTTCCCGTCCTTCATCCACT	85	XM_038704976.1
CAT-R	CAGGCTCCAGAAGTCCCACA		
GPx-F	CCCTGCAATCAGTTTGGACA	94	XM_038697220.1
GPx-R	TTGGTTCAAAGCCATTCCCT		
AKT-F	ATGGACTCCTCTCCAGACCC	164	XM_038729217.1
AKT-R	TTCATGGCGTACTAGCGTCC		
S6K1-F	GCCAATCTCAGCGTTCTCAAC	156	XM_038713349.1
S6K1-R	CTGCCTAACATCATCCTCCTT		
4EBP1-F	AGCAGGAACTTTCGGTCATA	168	XM_051877810.1
4EBP1-R	GTCAATGGGCAGTCAGAAGA		
mTOR-F	TCAGGACCTCTTCTCATTGGC	208	XM_038723321.1
mTOR-R	CCTCTCCCACCATGTTTCTCT		
PI3K-F	AAGACCTTCCTCATCACGAC	154	XM_038733025.1
PI3K-R	CCTTCCACTACAACACTGCA		
AMPKα-F	CACATGAATGCCAAGATTG	131	XM_038734014.1
AMPKα-R	GGACCAGCATATAACCTTC		
ATF6-F	CAGGACGAAGTGCTTAGAGTT	175	XM_038716053.1
ATF6-R	AGAGTAATGGACGGTCACAAT		
XBP1-F	ACACCCTCGACACGAAAGA	213	XM_038703562.1
XBP1-R	AGAATGCCCAGTAGCAAATC		
IRE-F	ACGGACCAATCGTGAGAC	244	XM_042730942.1
IRE-R	CGGGAGGTGAAGTAGGAG		
GRP78-F	TTGCCGATGACGACGAAA	180	XM_038733280.1
GRP78-R	CAATCAGACGCTCACCCT		
CHOPα-F	GATGAGCAGCCTAAGCCACG	153	XM_038701049.1
CHOPα-R	AACAGGTCAGCCAAGAAGTCG		
CHOPβ-F	GTATCTTCATTACCAGTCCACCAG	156	XM_046055812.1
CHOPβ-R	AGGCGTTTCTTTGCTTTCC		
BCL-2-F	CGCCATCCACAGAGTCCT	145	XM_038717982.1
BCL-2-R	CCGGAACAGTTCGTCTATCACC		
TRAF-F	CTGCCAAACCTTAATCCTT	261	XM_038734172.1
TRAF-R	ACAGACTTACAGCCCACTTC		
Caspase3-F	GCTTCATTCGTCTGTGTTC	98	XM_038713063.1
Caspase3-R	CGAAAAAGTGATGTGAGGTA		
Caspase8-F	GAGACAGACAGCAGACAACCA	195	XM_046051832.1
Caspase8-R	TTCCATTTCAGCAAACACATC		
Caspase9-F	CTGGAATGCCTTCAGGAGACGGG	125	XM_038734900.1
Caspase9-R	GGGAGGGGCAAGACAACAGGGTG		

Hif-1α = hypoxia inducible factor 1*α*; *Nrf2* = nuclear factor erythroid 2-related factor 2; *Keap1* = kelch-like ECH-associated protein 1; *SOD* = superoxide dismutase; *CAT* = catalase; *GPx* = glutathione peroxidase; *AKT* = protein kinase B; *S6K1* = ribosomal protein s6 kinase polypeptide 1; *4EBP1* = Eukaryotic initiation factor 4E-binding protein 1; *TOR* = target of rapamycin; *PI3K* = Phosphoinositide 3-Kinase; *AMPKα* = adenosine 5′-monophosphate (AMP)-activated protein kinase *α*; *ATF6* = activating transcription factor 6; *XBP1* = X-box-binding protein 1; *IRE* = inositol-requiring enzyme; *GRP78* = glucose regulated protein 78; *CHOPα* = DNA damage-inducible transcript 3 protein *α*; *CHOPβ* = DNA damage-inducible transcript 3 protein β; *BCL-2* = B-cell lymphoma-2; *TRAF* = tumor necrosis factor receptor-associated factors; C*aspase3* = cysteine-aspartic acid protease 3; *Caspase8* = cysteine-aspartic acid protease 8; *Caspase9* = cysteine-aspartic acid protease 9.
